# Spatial Ecology of the American Crocodile in a Tropical Pacific Island in Central America

**DOI:** 10.1371/journal.pone.0157152

**Published:** 2016-06-09

**Authors:** Sergio A. Balaguera-Reina, Miryam Venegas-Anaya, Andrés Sánchez, Italo Arbelaez, Harilaos A. Lessios, Llewellyn D. Densmore

**Affiliations:** 1 Department of Biological Sciences, Texas Tech University, Lubbock, TX 79409–3131, United States of America; 2 Smithsonian Tropical Research Institute, Apartado Postal 0843–03092, Balboa, Ancón, Republic of Panama; 3 Facultad de Biología, Universidad del Quindío, Armenia, Colombia; 4 Facultad de Biología Marina, Universidad de Bogotá Jorge Tadeo Lozano, Bogotá, Colombia; U.S. Geological Survey, UNITED STATES

## Abstract

Conservation of large predators has long been a challenge for biologists due to the limited information we have about their ecology, generally low numbers in the wild, large home ranges and the continuous expansion of human settlements. The American crocodile (*Crocodylus acutus*) is a typical apex predator, that has suffered from all of these characteristic problems, especially the latter one. Humans have had a major impact on the recovery of this species throughout its range, even though most of the countries it inhabits have banned hunting. The last decade has made it clear that in order to implement sound conservation and management programs, we must increase our understanding of crocodile spatial ecology. However, in only two countries where American crocodiles have telemetry studies even been published. Herein we have characterized the spatial ecology of *C*. *acutus* on Coiba Island, Panama, by radio-tracking (VHF transmitters) 24 individuals between 2010 and 2013, to determine movement patterns, home range, and habitat use. We have then compared our findings with those of previous studies to develop the most comprehensive assessment of American crocodile spatial ecology to date. Females showed a higher average movement distance (AMD) than males; similarly, adults showed a higher AMD than sub-adults and juveniles. However, males exhibited larger home ranges than females, and concomitantly sub-adults had larger home ranges than juveniles, hatchlings, and adults. There was an obvious relationship between seasonal precipitation and AMD, with increased AMD in the dry and “low-wet” seasons, and reduced AMD during the “true” wet season. We found disaggregate distributions according to age groups throughout the 9 habitat types in the study area; adults and hatchlings inhabited fewer habitat types than juveniles and sub-adults. These sex- and age-group discrepancies in movement and habitat choice are likely due to the influences of reproductive biology and Coiba’s precipitation cycle. Juveniles also showed distinct movement patterns and home ranges; however, with sexual maturation and development, these behaviors became more characteristic of adults and sub-adults. Ours is one of a very small number of studies that will allow future management and conservation planning to be based on the comprehensive integration of the spatial ecology of a Neotropical crocodylian apex predator.

## Introduction

Large predator management and conservation has been a difficult challenge for the scientific community for some time [1] due to the lack of carefully collected ecological information that allows a general understanding of their movements and relationships with their habitats. In addition, the continuous expansion of human settlements often reduces both suitable habitat and decreases the abundance of these species [2], making them more difficult to track and accurately evaluate their relationship to the environment. The American crocodile (*Crocodylus acutus*) is no exception to this trend despite the fact that it has the largest range of any of the Neotropical crocodiles, inhabiting North, Central, and South America on both coasts as well as several oceanic islands in the Caribbean and Pacific [3,4]. Even after more than 30 years of banned hunting across most of its range, *C*. *acutus* is still one of the most threatened of all New World crocodile species (along with the Cuban and the Orinoco crocodiles [5,6]). It has suffered from over-hunting and a general reduction of optimal habitat across its entire range during the last century [3,7,8]. *C*. *acutus* is currently catalogued as *Vulnerabl*e in the International Union for Conservation of Nature (IUCN) RedList [9] and as *Endangered* in the Panamanian Red List (resolution No. AG–0051–2008). Panama occupies a central position in the species range, but the current state of knowledge of Panamanian populations is poor, and concerns about its conservation in many parts have been raised [10,11].

Coiba National Park (CNP) is the largest marine protected area in Panama. It is included as a World Heritage Site due to its significant biodiversity and overall biological and ecological importance [12]. It is located in the Gulf of Chiriqui on the Pacific coast, being part of the Tropical Eastern Pacific Marine Corridor along with several other island groups, including Coco (Costa Rica), Galapagos (Ecuador), Malpelo, and Gorgona (Colombia) [13]. CNP is comprised of Coiba Island (the largest island on the Pacific side of Central America) and 38 minor islands and rocky islets [12].

One of the largest gaps in knowledge that we believe contributes to the threatened status of the American crocodile across its entire range (and particularly in Panama), is the absence of data from any long-term studies on its movement patterns, home ranges, and habitat use [8,11]. Telemetry has proven to be very valuable in clarifying movement patterns and home ranges, generally providing greater accuracy than classic methods like mark-recapture [14]. It has also contributed to our understanding of the dynamic and seasonal patterns of habitat use by the generally recognized different crocodylian life-history stages [14]. Currently, in only two of the 18 countries that the American crocodile inhabits have there been published telemetry studies on this species. In the United States, nine adults (two males and seven females) and one sub-adult (female) were radio-tracked (VHF transmitters) by boat and airplane from 1978 to 1981 in the southern tip of Florida between Key Largo and Cape Sable [15,16]. Another study (also in Florida) involved four adults, two sub-adults, and three juveniles, which were radio-tracked in the vicinity of the Turkey Point Power Plant [17]. In Panama, 10 juveniles (nine 10-month old animals and one 22-month old animal) were followed using radio-telemetry in Gatun Lake [18]; also five sub-adults (one female and four males) were tagged (VHF Transmitters) and followed from 2010 to 2011 on Coiba Island [19,20]. Despite the critical importance of spatial ecology to understand crocodylian life history, ecological parameters such as home range behavior and dispersal patterns have received poor (to no) attention in most countries where *C*. *acutus* occurs.

To date, telemetry analyses have been reported for ten out of the 24 species of crocodylians (other than the American crocodile) including: *Caiman yacare* (also known as *Caiman crocodilus yacare*) [21,22], *Melanosuchus niger* [23], *Alligator mississippiensis* [24–30], *Alligator sinensis* [31,32], *Paleosuchus trigonatus* [33], *Tomistoma schlegelii* [34], *Crocodylus johnstoni* [14], *Crocodylus niloticus* [35,36], *Crocodylus porosus* [37–39], and *Crocodylus intermedius* [40,41]. It is also notable that for most of the species there have only been one or two descriptive studies, involving low numbers of animals, which were followed for short time periods. Actual spatial ecology research has only been accomplished in the American alligator (*A*. *mississippiensis*), in studies performed in the United States [42].

Currently, determination of movement patterns, habitat use, and overall home ranges are the major research priorities identified for American crocodiles in Panama [11]. These were also designated as priorities across its entire range, along with development of conservation programs in three countries, Peru, Ecuador, and Colombia [3]. By understanding these aspects of their biology and spatial ecology, we will be able to develop plans to reduce potential conflicts with humans and protect this species. The United States (Florida), Cuba, and Costa Rica currently seem to have what apparently are very healthy populations [3]. However, only in Florida has the spatial ecology of the America crocodile been characterized in such a way to allow comprehensive management and modelling restoration processes to be developed and implemented, that include habitat (using American crocodile as a species indicator), *C*. *acutus* populations, and people [43]. This same type of research approach needs to be undertaken throughout *C*. *acutus*’ distribution in order to develop conservation plans that are applicable range-wide. Towards that end, we have assessed the spatial ecology of the American crocodile in Coiba National Park between 2010 and 2013. Data from 2010 and 2011 came from a previous pilot study done by our team [19,20]; these were reanalyzed along with new data collected during 2013. Our efforts also allowed us to determine the dynamic and seasonal patterns of habitat use by different life-history stages and by sex on insular habitats, which to our knowledge has not been described and/or published before now.

## Materials and Methods

**Fig 1:**
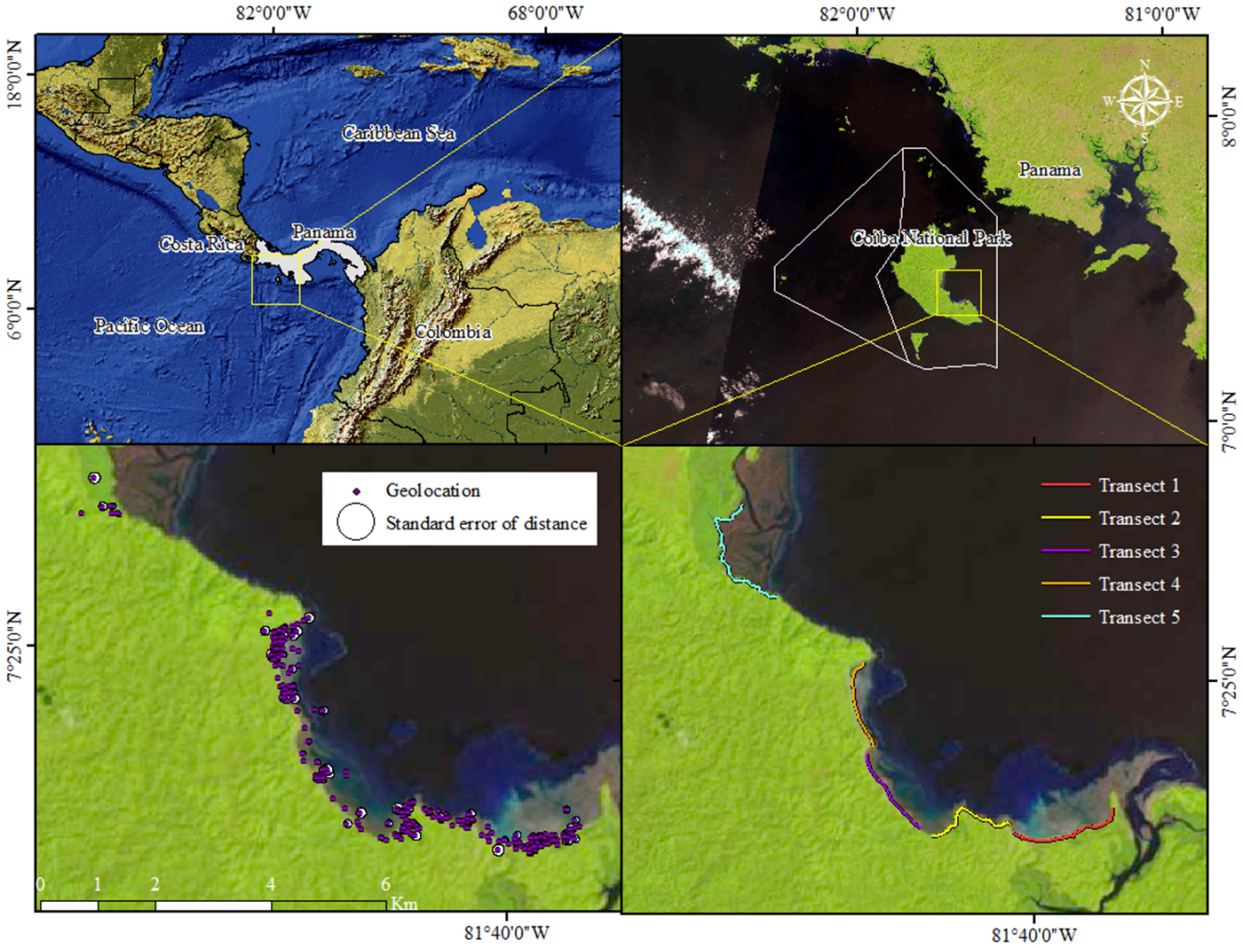
Study area. Location of the Coiba National Park along the Pacific coast of Panama highlighting transects followed throughout this study.

Early on in the fieldwork phase of the study, we did nocturnal spotlight survey transects [44] on foot across the coastal zone, creeks, streams, mangrove and riparian forests in southeastern Coiba (Fig 1), capturing any individuals we encountered. We captured and tagged a total of 24 individuals, which were monitored for a period ranging from 7 to 10 months (four individuals followed from September 2010 to April 2011-documented in our previous work [19,20] and 21 from February to December 2013. One animal was tagged during both 2010 and 2013). All individuals were sexed (via cloacal probing), measured (total length-TL, snout-ventral length-SVL, head length-HL, head width-HW, and weight-W), marked by notching scales in the tail, tagged with a transmitter (Telenax^®^ VHF models TXE-311BR and TXE-304BR), and returned and released at the original capture. We classified them and analyzed the data using age groups (juvenile TL 30–90 cm, sub-adult TL 91–180 cm, and adult TL > 180 cm) [45].

Based on the information collected using these transects, we report the geolocations of 24 individuals and the standard error of distance (which is the “uncertainty” component of the data). A transmitter was attached to the neck of each individual using wire wrapped around the transmitter, over the cervical scutes and below the osteoderms. Each transmitter had a unique frequency to allow unambiguous identification of every individual. We did not attach radio-telemeters to hatchlings because the size of the equipment was not appropriate. Instead, we separately reanalyzed hatchling (TL < 30 cm) and early juvenile (TL < 50 cm) data that we had collected in the same area in a previous study where we assessed the reproductive ecology and hatchling growth rates of *C*. *acutus* using mark-recapture from April to December 2013 [4]. This allowed us to cover all of the major life-history stages. The hatchling/early juvenile data were not included in the movement pattern estimations, but were incorporated into the home range, utilization distribution, and habitat use evaluations.

Animals were monitored daily; transects were followed by foot with stations for reception every 50 m across the shore area (Fig 1). In these transects, using a three-element-Yagi antenna and a receptor (Telenax^®^ RX-TLNX), we determined the geo-reference (from the station where the single signal was detected) and the heading per frequency/individual. If an animal was not detected anytime over a period of one week, we expanded the search area (by foot or by boat) until it was recorded again and included the new area in the expanded transect. Whenever possible, we did a visual inspection of the animal and the transmitter, collecting the geolocation data where the animal was located. After 12 hours of no detectable movement by the transmitter, a ‘mortality’ sensor inside each transmitter was activated, and reported a double signal, differing clearly from the single signal emitted by the transmitter when animal is still alive and the transmitter is attached to its neck. In these cases, an intensive search was carried out to determine whether the animal had died or simply detached of the transmitter.

All collected data were analyzed in ArcGIS 10.2.2 [46]. The triangulation estimates were made using a mathematical model [19,20], which was generated in ModelBuilder [46] and modified for this project. It estimates the intersection points based on the geo-reference (the exact location where an animal was detected by the researcher using the receptor), the heading from where the signal came, and a 1 km line (determined by relying on previous field experience using the same type of tags in mangrove areas). With this information, we estimated the mean geographical center of those areas based on the intersections constructed from the average distances [x and y] among the three sets of coordinates, thus determining the geolocation (mean geographical center) for each crocodile. We also generated an uncertainty estimate for each of these geolocations by determining the standard error of distance, which measures the degree to which the three intersections are distributed around the mean geographical center [46]. We only considered data with three intersections as the minimum amount of information required to estimate the standard error of distance. Geolocations with uncertainty values over 100 m (≥ 0.03 km^2^ circular area) were treated as outliers and were deleted from the calculations.

We filtered these final geolocations using the ArcMET extension [47] of ArcMap [46], and included the hatchling/early juvenile geolocations derived from our previous study [4], based on three criteria: (1) temporally non-overlapping positions (minimum time separation between geolocations of 30 seconds), (2) sequential, spatially non-intersecting positions (minimum distance separation between geolocations of 50 cm), and (3) a threshold speed (maximum speed = 12 km/h). We chose the first two criteria to avoid temporal and spatial overlaps and the third was based upon recorded crocodile speed estimates documented by a colleague [48]. From these filtered data, we calculated each animal’s trajectory, taking into account the Average Time between Geolocations (ATG), Average Movement Distance (AMD), and Average Movement Speed (AMS) per individual, sex, and age group. These results were analyzed statistically using Infostat and R software [49,50] to determine whether the variation in AMD was equal or differed among months, precipitation seasons (based on average historical precipitation estimates from 1971–2014) [51], and groups. Shapiro-Wilk tests were performed to determine the normality of the data and Kruskal-Wallis tests were run to analyze its variability. Dunn's-test for independent samples with a Bonferroni adjustment of p-values was used to determine pairwise differences of mean ranks, when Kruskal-Wallis tests were significant (p < 0.05). We estimated Conspecific Proximity (CP) [47] to determine the inter-crocodile distances based on a temporal range of 6 hours. This interval was chosen based on the tidal cycles reported in the study area.

We assessed spatial autocorrelation for the data both by individual and by hatchling/early juvenile [4] based on the time lag between geolocations using Moran’s I analysis [52] in ArcGIS [46]. From these results, for each individual we estimated the Minimum Convex Polygon (MCP) [53], the Kernel Density Estimation (KDE) [54], and the Local Convex Hull—Adaptive (aLoCoH) [55] using isopleths 50 (core) and 95 (the contour that captures 50 or 95% of the data related to the distance between them) to determine the home range and the utilization distribution of American crocodiles in the study area. These data were analyzed as a whole (all individuals) as well as by sex and by age groups. We included MCP and Kernel core estimates for comparison purposes because they have been commonly used in the literature. We also included Local Convex Hull because it estimates home range in a more adaptive way [55]. In the KDE analysis, we estimated the optimum smoothing parameter (h-ref) based on the spatial variance of the input geolocations [56]. In the aLoCoH case, we set the distance threshold based on the maximum displacement by individuals estimated within the area. Pixel resolution in the case of KDE was set up based on the average standard error of distance (geolocations uncertainty).

We performed a site fidelity test per individual (bootstraping it 10,000 times) using the reproducible home range (rhr) package in R [57]. We estimated the mean squared distance from center of activity (MSD) and the linearity index (LI), determining where the critical threshold was located (α = 0.05). Site fidelity was recognized when the observed area that an animal used was smaller than the area used if an individual’s movement was random [58].

Our primary hypothesis was that movement patterns and home ranges in American crocodiles are influenced by the availability of food resources as reflected by the suitability of water sources (seasonality), beaches for nesting, and land-cover around bodies of water (mangrove and riparian forest) for different age groups and sexes. This hypothesis was tested against the null hypothesis of a random pattern in those spatial variables in the study area.

“Size class” has been a common way to report data in demographic studies in American crocodiles due to its practicality [59]. Because of this, we also provide all calculations using this classification method as supplementary material (S1–S3 Tables; S1 Fig) with the main idea to generate a referent point for those who use this classification method.

Geolocations were buffered by creating a polygon around each point based on a defined distance on the basis of the estimated uncertainty and examined as to whether it overlapped land-cover, coral reefs, and river layers as determined from the Coiba Natural Park (CNP) management plan (created using the 2006 Advanced Spaceborne Thermal Emission-ASTER imagery data) [12]. We adjusted the layer boundaries using imagery provided by ESRI (Environmental Systems Research Institute) in ArcGIS [46] and generated habitat use estimates according to sex and age group. We also buffered geolocations based on the AMD by individual to determine the land-cover that the animals were inhabiting. We report the accuracy of sample means using a standard deviation of ± 0.1 (SD) and in those cases where this value was larger than the mean due to the natural skew of the data we used minimum and maximum values (min-max).

## Results

We surveyed the study transects by foot 245 times collecting data from individuals tagged with VHF transmitters (~ 15 km along the shore line). From September 2010 to April 2011, we surveyed the transects 101 times, monitoring four animals [19,20]. From February to December 2013, we surveyed the transects 144 times, monitoring 21 animals. One animal (ID84) was captured and tagged twice (during 2010 and 2013) giving a total number of 24 different individuals that were studied. Over the three-year span, we collected a total of 742 geolocations (676 from telemetry and 66 from sightings, capture and release geolocations) from 14 males (10 sub-adults and 4 juveniles) and 10 females (5 adults and 5 sub-adults; Table 1). Sizes ranged in females from 96 cm to 256 cm (TL); in contrast male sizes ranged from 76 cm to 167 cm. However, we did not find a significant difference between TL and sex (K-W χ^2^ (1) = 3.15, p = 0.075, α = 0.05).

**Table 1:** Trajectories, movement distances, and speed of the American crocodile.

ID	Sex	Age Group	TL (cm)	N	TAG (h) max-min	AMD (m) max-min	AMS (m/h) max-min
404	F	Adult	256	13	141 (3–1,320)	907 (8–5,607)	24 (0–240)
417	F	Adult	210	3	258 (16–658)	396 (3–1,055)	4 (0–11)
146	F	Adult	219	16	329 (8–1,565)	270 (4–973)	9 (0–116)
405	F	Adult	223	27	254 (4–1,653)	171 (17–657)	6 (0–50)
441	F	Adult	218	14	334 (23–1,585)	138 (2–580)	2 (0–5)
412	M	Sub-adult	167	9	460 (16–1,987)	1132 (242–4,337)	7 (1–27)
402	M	Sub-adult	123	17	269 (7–2,375)	960 (23–2,899)	29 (0–338)
476	M	Sub-adult	150	5	632 (41–2,375)	470 (107–1,224)	4 (0–12)
105	M	Sub-adult	154	34	187 (4–1,251)	401 (3–3,787)	3 (0–19)
403	M	Sub-adult	129	19	308 (7–3,833)	320 (4–1,441)	6 (0–25)
414	M	Sub-adult	123	19	285 (4–1,337)	162 (48–449)	4 (0–31)
84	M	Sub-adult	124	63	104 (2–1,228)	81 (3–463)	3 (0–42)
			145	41	144 (2–1,094)	241 (5–775)	10 (0–64)
128	M	Sub-adult	92	2	31 (14–47)	1107 (335–1,878)	31 (23–40)
400	F	Sub-adult	100	10	227 (5–1,375)	753 (18–2,013)	9 (1–30)
530	F	Sub-adult	96	8	302 (9–1,618)	727 (77–1,287)	36 (1–149)
419	M	Sub-adult	108	3	390 (39–633)	684 (187–1,033)	8 (0–22)
411	F	Sub-adult	98	16	267 (3–2,455)	562 (23–5,807)	12 (0–104)
100	F	Sub-adult	109	34	183 (2–1,265)	362 (21–1,169)	15 (0–260)
407	F	Sub-adult	113	20	216 (7–1,680)	91 (17–399)	3 (0–16)
82	M	Sub-adult	93	43	128 (1–1,699)	35 (2–480)	3 (0–36)
416	M	Juvenile	76	6	187 (14–356)	607 (370–1,207)	8 (1–26)
410	M	Juvenile	76	12	50 (6–161)	163 (3–396)	10 (0–50)
135	M	Juvenile	87	14	145 (11–497)	139 (4–367)	2 (0–6)
122	M	Juvenile	90	23	193 (5–1,315)	128 (10–553)	7 (0–91)
**Average**			135.2		241 (10–1,414)	280 (76–1,192)	7 (2–63)

Number of path trajectories (N), time average between locations (TAG), average movement distance (AMD), and average movement speed (AMS) for each Coiba Island individual followed (ID), classified by sex (S; Female F; Male M) and age group. Individual 84 was monitored in two periods: the first line in 2010–2011, the second line 2013.

The uncertainty value of the geolocations was 38.0 ± 54.8 m, ranging from 0.1 m to 389.2 m with a median value of 18.6 m (n = 472 without including sightings, capture, and release points). The distances covered a circular area of 4,536.4 ± 9,434.3 m^2^ (See lower left panel Fig 1). The majority of geolocations had an uncertainty value of ≤ 26 m (60%) and less than 9% of the data had values over 100 m. The uncertainty average without outliers (≥ 100 m) was estimated to be 24.4 ± 23.1 m (covering a circular area of 1,870.3 ± 1,676.3 m^2^). After filtering the data and including capture and release points, we recorded a total of 498 geolocations. In the case of hatchlings (< 30 cm TL) and early juveniles (< 50 cm TL), we used 142 geolocations collected in the period of reproduction in 2013 from our earlier study [4].

The average movement distance (AMD) by an individual was 280 m (ranging from 76 to 1192 m) (Table 1). The highest and lowest AMD values were in two sub-adult males (ID412 and ID82, respectively). The highest AMD value among females was recorded in an adult (ID404) and the lowest in a sub-adult (ID407; Table 1). A sub-adult male individual (ID402) had the highest average movement speed (AMS) value recorded in the study (0.34 km/h).

**Fig 2:**
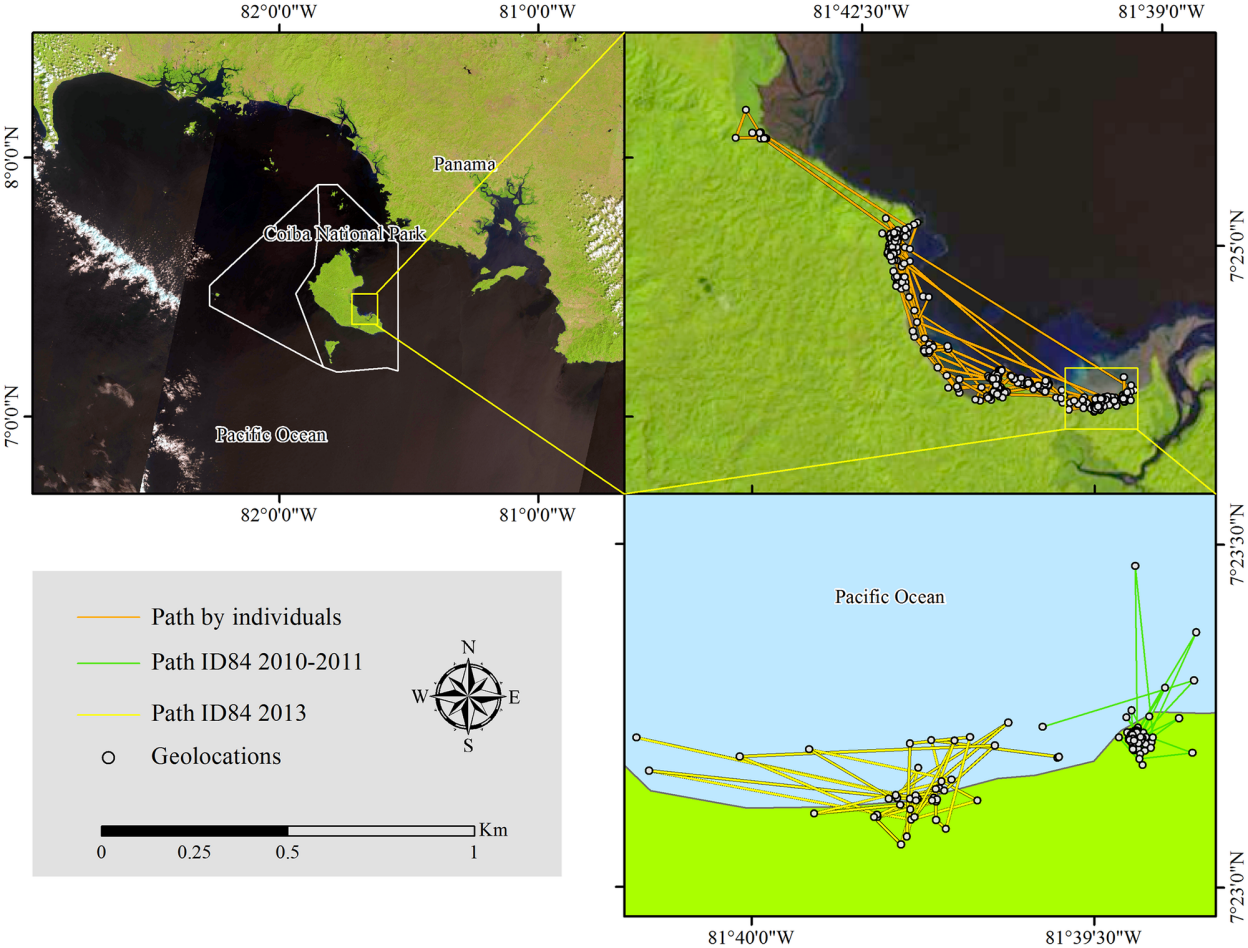
Trajectory movements. Trajectory movements of all the American crocodile individuals followed between 2010 and 2013 in Coiba Island, highlighting the differences in the trajectory of individual ID84, which expanded its movement area without overlapping with the previously estimated one.

We determined, on average, two geolocations (TAG) from the same individual every 10 days. In the case of ID84, the AMD value increased from 2010 to 2013, as did the TAG (Table 1). Interestingly, paths of ID84 never overlapped between 2010 and 2013, indicating that he was using different zones in the same area (Fig 2). On average, females moved longer distances than males and adults moved longer distances than sub-adults and juveniles, respectively (Table 2). It is important to highlight that the absence of adult males could reduce the AMD in that group, suggesting that males can move longer distances than recorded in this study.

**Table 2:** Trajectories, movement distances, and speed of the American crocodile by age group and sex.

Groups	# of individuals	N	TAG (h) max-min	AMD (m) max-min	AMS (m/h) max-min
Adult	5	59	250 (3–1,653)	372 (3–5,607)	10 (0–240)
Sub-adult	15	356	204 (1–3,837)	311 (2–5,807)	24 (0–2,739)
Juvenile	4	55	149 (5–1,315)	191 (3–1,207)	15 (0–253)
Female	10	161	242 (2–2,455)	375 (2–5,807)	29 (0–2,739)
Male	14	309	184 (1–3,833)	268 (1–4,336)	17 (0–661)

Number of path trajectories (N), time average between geolocations (TAG), average movement distance (AMD), and average movement speed (AMS) followed by American crocodiles in Coiba Island per age group and sex.

We did find significant differences in the AMD by individuals (K-W χ^2^ (23) = 132.04, p = <0.001, α = 0.05), years (K-W χ^2^ (2) = 47.97, p = <0.001), months (K-W χ^2^ (11) = 31.40, p-value = 0.001, α = 0.05), and sex (K-W χ^2^ (2) = 9.38, p-value = 0.010), but not by age groups (K-W χ^2^ (2) = 0.67, p = 0.716). The pairwise comparisons using Dunn's-test shows that on average the majority of individuals did not move significantly differently, with the exceptions of ID82 with respect to 16 individuals, and ID84 and ID407 with respect to 2 individuals (Table 3). On average, individuals moved significantly differently through years with the exception of 2010 and 2011 (p = 0.410). Finally, individuals did not move significantly differently among months with the exception of May and October (p = 0.018).

**Table 3:** Pairwise comparisons using Dunn's-test for independent samples.

	ID82		ID412	ID530		Dry season	High-wet season
**ID84**	<0.001	**ID84**	0.016	0.038	**High-wet season**	0.015	
**ID100**	<0.001	**ID407**	0.026		**Low-wet season**	0.013	<0.001
**ID105**	0.001						
**ID146**	0.001						
**ID400**	<0.001						
**ID402**	<0.001		**2010**	**2011**		**Paternal care**	
**ID403**	0.034	**2013**	<0.001	<0.001	**Nesting**	0.039	
**ID405**	<0.001				**Courtship and Mating**	0.002	
**ID410**	0.019						
**ID411**	0.002						
**ID412**	<0.001						
**ID414**	0.001		**May**				
**ID416**	<0.001	**October**	0.018				
**ID419**	0.037						
**ID476**	0.008						
**ID530**	<0.001						

Pairwise comparisons using Dunn's-test for independent samples between individuals (ID#), years, months, precipitation seasons, and reproductive ecology in Coiba Island. We only report data with significant pairwise comparisons values (p-value = <0.05).

**Fig 3:**
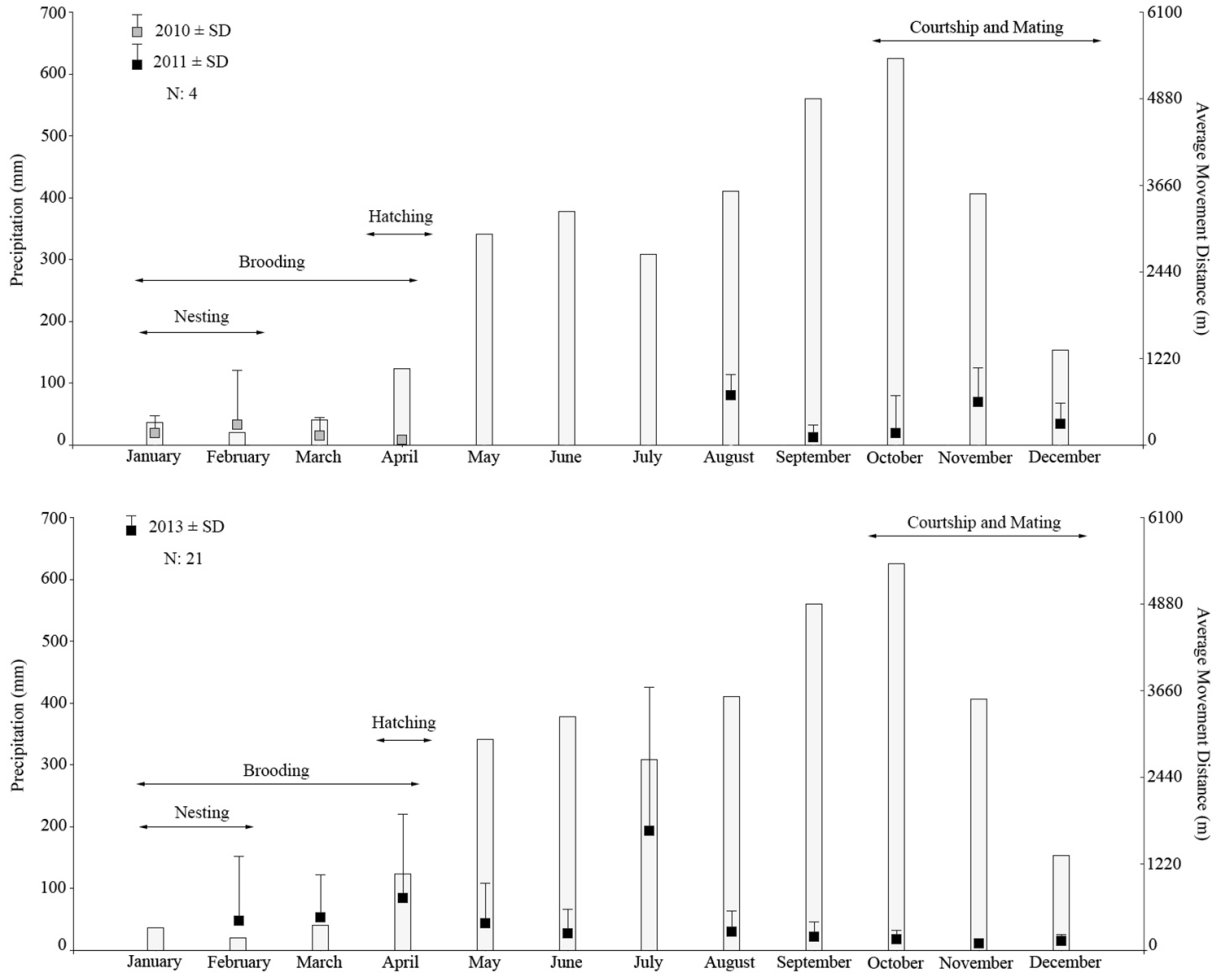
Average movement distances by years and average precipitation. Average movement distances (AMD) of the American crocodile in Coiba Island from 2010 to 2013 related to the average historical precipitation (1971–2014) in the area and the reproductive ecology reported for our previous work [4]. Note the AMD increase in the dry season and decrease in wet season.

**Fig 4:**
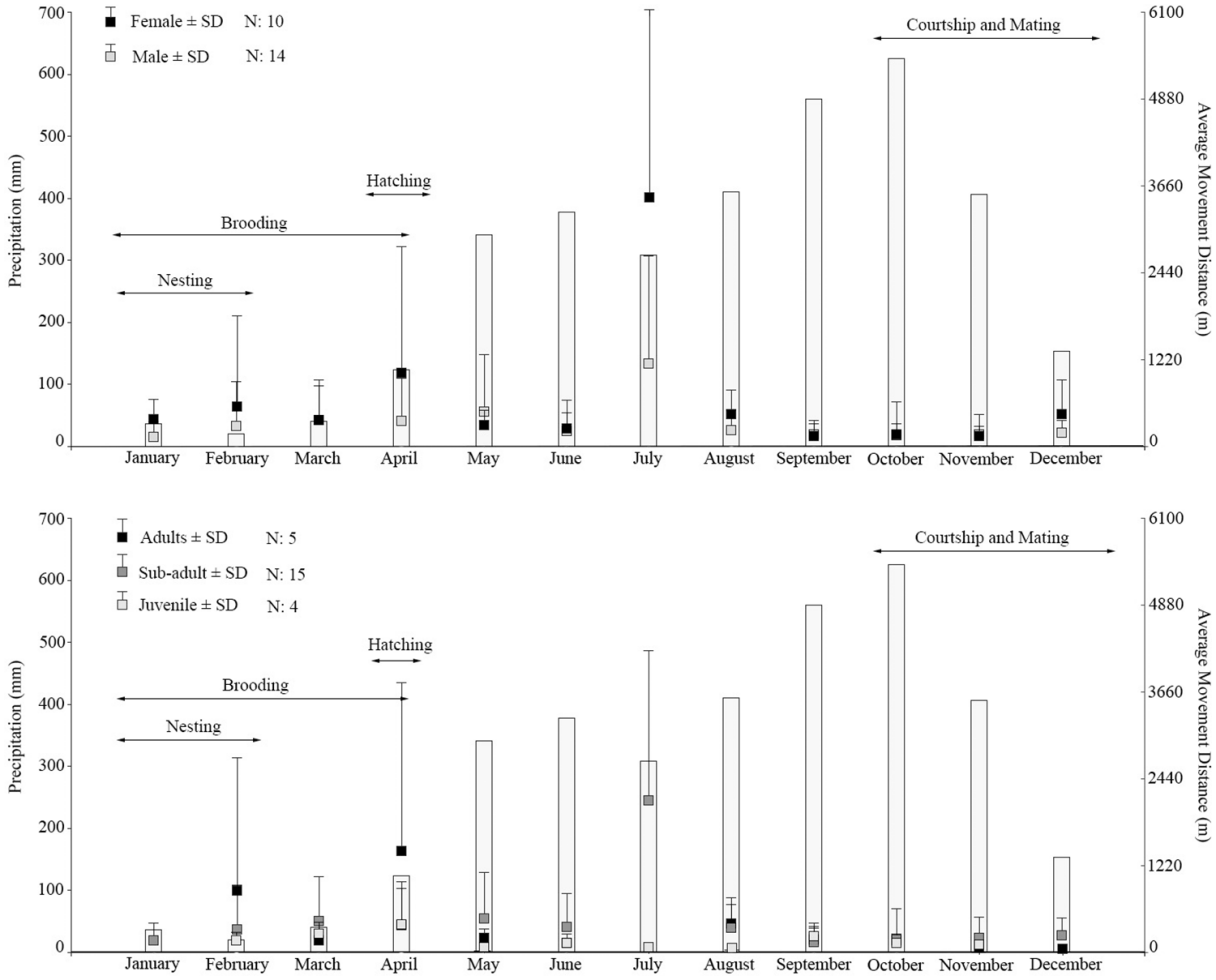
Average movement distances by sex and age group and the average precipitation. Average movement distances (AMD) per sex and age group of the American crocodile in Coiba Island related to average historical precipitation and reproductive ecology. Changes in AMD between seasons are clearer in females, adults, and sub-adults.

We found significant differences between AMD and precipitation season (K-W χ^2^ (2) = 23.88, p = <0.001; Fig 3) as well as between AMD and the reproductive behavior (K-W χ^2^ (5) = 18.60, p-value = 0.002). On average, individuals moved significantly differently between the high-wet season (September to November, 400–626 mm of precipitation per month) and dry season (December to April, < 200 mm), low-wet season (May to August, 300–400 mm) and dry seasons, and low-wet season and high-wet season (Table 3). Likewise, individuals moved significantly differently between nesting time (January) and parental care time (May and June) and between courtship and mating time (October to December) and paternal care time (Fig 4; Table 3).

Spatial autocorrelation analysis indicated that there was no autocorrelation among geolocations by individuals, either in all data or for just hatchlings/early juvenile, based on the time lag between geolocations for each individual (Z score: 0.07, 0.90, and -0.22; Moran’s I index: -0.00, 0.00, and -0.22; p > 0.05, respectively). The conspecific proximity (CP) within a temporally overlapping period of 6 h was on average 1,883.0 ± 2,121.3 m, ranging between 16.5 m to 10,055.1 m (N = 838). Individuals spent the majority of time (> 60% of events) farther than 500 m from each other; only 20% of geolocations were 200 m or less from one another. Females were found up to 200 m from males 25% of the time; juveniles were never this close to an adult, and were located at this distance from sub-adults up to 17% of the time, and up to 50% of the time from hatchlings (Table 4). In contrast, hatchlings were equal to or less than 200 m from sub-adults 4% of the time, whereas 50% of the time they were 200 m or less from adults. Finally, adults were up to 200 m from sub-adults 45% of the time (Table 4).

**Table 4:** Conspecific proximity among American crocodiles.

	N	Up to 200 m (%)	ACP (m)
Females to males	276	25	1,533.9 ± 1,578.9
Juveniles to adults	20	0	2,297.9 ± 1,659.3
Juveniles to sub-adults	109	17	1,246.5 ± 1,310.3
Juveniles to hatchlings	4	50	2,891.5 ± 2,843.9
Hatchlings to sub-adults	24	4	1,936.9 ± 1,866.0
Hatchlings to adults	4	50	117.4 ± 90.0
Adults to sub-adults	151	45	1,000.5 ± 1,594.0

Number of observations, percent of geolocations closer than 200 m, and average conspecific proximity (ACP) within a time overlap of 6 h estimated for 24 American crocodiles followed in Coiba Island from 2010 to 2013 divided by sex and age group.

**Fig 5:**
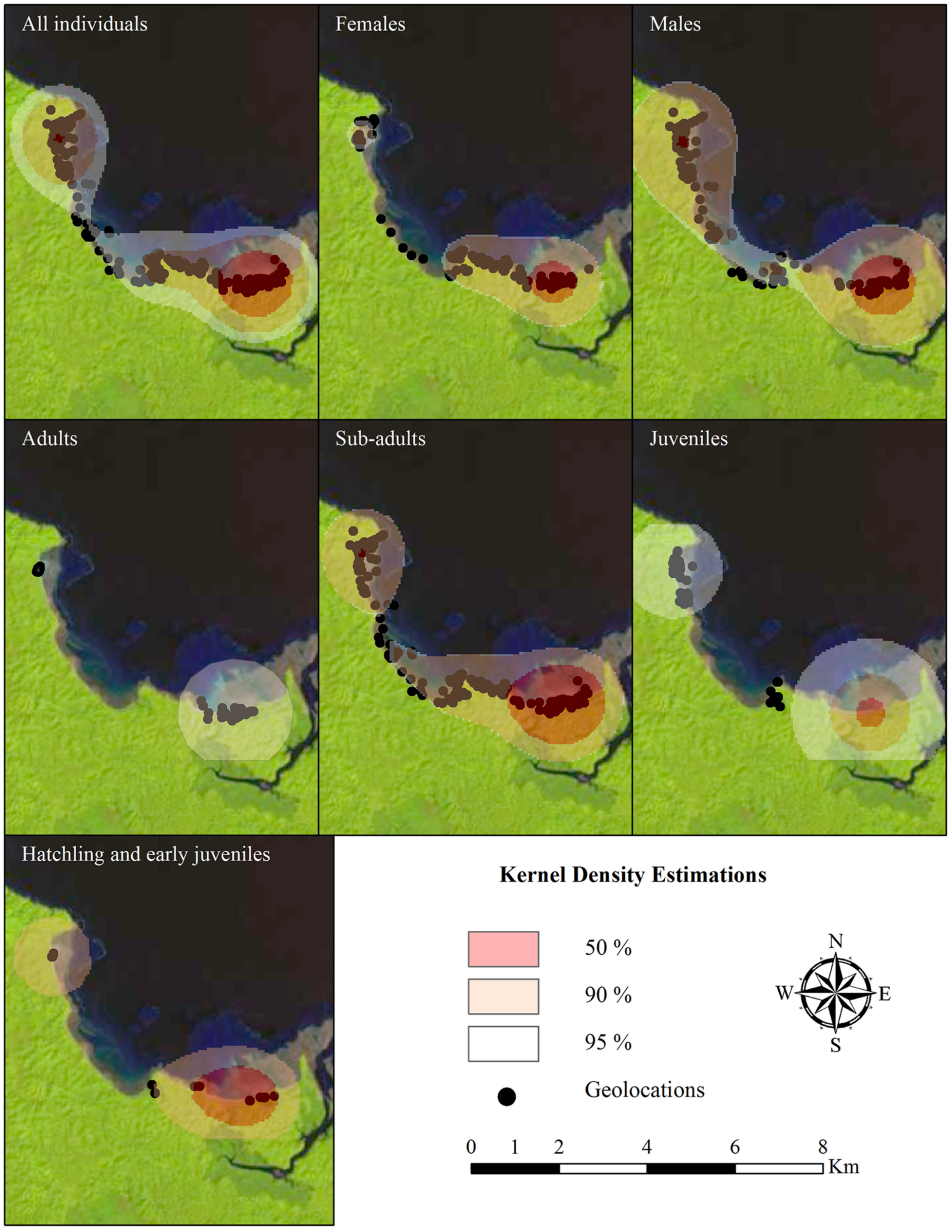
Home range and utilization distribution. Utilization distribution per group (sex and age group) and total geolocations using Kernel Density Estimation (KDE).

The MCP for the total data set was larger than the aLoCoH at 50 (core-use area) and 95% of the total area and the KDE at 50 but not at 95% of volume contours of the Kernel estimator of the data set (Table 5, Fig 5). According to the three methods, the home range and utilization distribution area in males was larger than females and in sub-adults larger than juveniles, hatchlings, and adults, with some variation in age groups at 50 MCP, KDE and aLoCoH (Table 5). In general, home ranges estimated using aLoCoH showed less variation among core-use areas and home range areas than KDE and MCP per individual, sex and age group being a more consistent estimator of core and home range areas than KDE and MCP.

**Table 5:** Home range and utilization distribution of the American crocodiles.

	50% (km^2^)			95% (km^2^)		
	MCP	KDE	aLoCoH	MCP	KDE	aLoCoH
Average by Ind.	0.0 ± 0.0	0.1 ± 0.4	0.1 ± 0.1	0.4 ± 0.6	1.7 ± 2.3	0.2 ± 0.2
Maximum	0.1	2.0	0.6	2.2	8.1	0.7
Minimum	0.0	0.0	0.0	0.0	0.0	0.0
Female	1.3	0.8	0.2	8.8	5.8	0.8
Male	6.1	1.6	0.4	10.4	15.2	2.6
Adult	0.2	0.0	0.1	2.5	5.0	0.3
Sub adult	2.6	3.2	0.4	10.4	12.8	2.6
Juvenile	0.3	0.3	0.2	2.2	12.1	0.3
Hatchling	0.4	2.0	0.2	4.3	8.1	0.3
All Individuals	4.6	2.0	0.3	10.5	14.5	2.2

Home range and utilization distribution of the American crocodiles on Coiba Island estimated via Minimum Convex Polygon (MCP), Kernel Density Estimation (KDE), and Local Convex Hull—adaptive (aLoCoH). These values were estimated from the average by individual + SD, reporting the maximum and minimum values obtained. Data for all individuals and averages divided by sex and age group are reported. Analyses were made including all data on all individuals and using isopleths at 50% and 95%.

We found site fidelity to a given area based on both indices (mean square distance-MSD and linearity index-LI) in the majority of animals (p-value = < 0.05; for IDs 84, 122, 128, 135, 146, 400, 403, 405, 410, 411, 412, 414, 419, and 530); only one animal did not exhibit this behavior (ID476). However, we obtained inconclusive results for nine individuals (p-value > 0.05 in the MSD or in the LI).

The animal that was followed twice (ID84) during two years (from April 2011 to April 2013), increased its AMD from 81.1 ± 10.3 m to 241.3 ± 14.6 m. Its home range increased from 0.0 km^2^ to 0.2 km^2^ (MCP) and from 0.0 km^2^ to 0.1 km^2^ (aLoCoH), both at 95% isopleth. This may indicate that movement patterns and home ranges are related and increase together in young animals, but when sexual maturation begins these types of behavior can be modified. We found no overlap in the paths of this animal with those of others, which might suggest that young animals will disperse when they are trying to find a suitable living area while simultaneously avoiding large crocodiles.

Taking geolocation uncertainty (25 m) into account, individuals were recorded in the sea 46% of the time and on the beach 19% of the time (including areas with vegetation and those without vegetation). They were also found in secondary forest 11% of the total time and in mangrove forest 10% of the total time, respectively, taking into account all the geolocations. The lowest presence of animals was in wet forest and any human-impacted areas (< 10% each). Males and females spent most of the time in the sea (42 and 47%, respectively) and on the beach (20 and 27%, respectively). Sub-adults were largely found in the sea (48%) more frequently than juveniles (45%), hatchlings (31%), or adults (23%). Hatchlings were recorded in a higher proportion in mangroves (14%) than sub-adults (11%), juveniles (8%), and adults (4%). In contrast, adults were more common on the beach (49%) than were hatchlings (30%), sub-adults (19%) or juveniles (14%).

Habitat use based upon total AMD also revealed a major presence of individuals in the sea (45%), secondary forest (25%), wet forest (13%), and mangrove forest (6%), with seemingly minor use of shrubland and pastures. This pattern was repeated in hatchlings, juveniles, sub-adults, males, females, and adults, except that mangroves were not used by the latter two. Sub-adults and juveniles seem to be more generalists using nine land-covers, two more than adults and hatchlings.

## Discussion

This project represents the largest telemetry study to date for *Crocodylus acutus*, in terms of number of animals tracked and time spent following them, and one of the largest for any crocodile species, covering all major life stages from hatchling to adult [45]. Previous studies in both Florida [15,16] and Panama [18–20] revealed some aspects about the spatial ecology of the American crocodile (home ranges and distance movements) according to sex or age groups in coastal ecosystems and inland water systems. Our analyses based on a larger database (considerably more animals and more geolocations through time), were able to produce a much more complete evaluation of how American crocodiles relate to the physical space that they inhabit in an insular area, including the effects of seasonal differences in precipitation on their movements and landscape use.

Triangulation is the most common way to estimate an animal's location [60]. However, most researchers have treated radio-telemetry data as exact points, neglecting to estimate geolocation uncertainty [61]; this affects the accuracy of home range, utilization distribution area, and habitat use pattern estimates. Previous studies on this species [15–20] have treated geolocations as exact data ignoring the measurement error; this resulted in serious underestimation of total variation in the data. This problem also exists in other studies of crocodiles [27,37,62], with implications on the results and conclusions generated depending on which analyses were performed [63]. Therefore, we used a spatial method (standard error of distance) to estimate the uncertainty of the triangulation technique on *C*. *acutus* (24.4 ± 23.0 m), allowing us to reduce the bias in our estimates of home range, utilization distribution, and habitat use. It also allowed us to define and filter outliers, reducing noise in the dataset, and increasing the accuracy of our analyses.

We found patterns in distance movements, home ranges, and utilization distribution by sex and age groups. Females showed a higher AMD than males and adults had a higher AMD than sub-adults and juveniles. Nevertheless, the absence of adult males in the present study may imply that the higher movement detected in adults could be because they were adults or they were all females. Thus, future studies should include adult males to test these findings. Regardless, overall, the larger animals had greater average movements than smaller ones. However, long-distance movement did not always mean larger home range area. We did find that males in general had larger home ranges and utilization distribution areas than females (regardless the absence of adults in the present study); similarly, sub-adults (females and males) displayed larger values in these two attributes than juveniles, hatchlings, and adults. Thus, sub-adults had the largest home ranges and utilization distributions followed by juveniles, whereas adults and hatchlings showed the smallest home ranges and utilization distributions. Based on these data, we found that females seemed to move long distances around one particular area (in our case, Playa Blanca and El Maria beaches), always coming back to or being around beaches (nesting areas), whereas males moved relatively short distances in a wider area (Fig 5). We also found that juveniles and sub-adults had larger home ranges than adults and hatchlings, spreading through these larger areas in relatively short movements. The kinds of dissimilarity in home range size and movement patterns observed in this study of *Crocodylus acutus* have been reported in other crocodylians, including *C*. *niloticus* [35], *C*. *johnstoni* [14], *Paleosuchus trigonatus* [33], and *C*. *porosus*, and thus, appear to be common in crocodiles [37].

We found a relationship between seasonal precipitation, reproductive ecology behaviors reported in the area [4], and the AMD, which were statistically significant based on both Kruskal Wallis and post-hoc Dunn’s tests. An individual’s average movement distance and its variation were higher during the dry and ‘low-wet’ season (from December to April and May to August) and lower during the true “wet” season (September to November). This pattern was clearly observed by both sex (mainly in females) and age groups (mainly in adults and sub-adults; Fig 4). All individuals’ movements analyzed pairwise per precipitation season as well as per nesting, courtship and mating, and paternal care times were significantly different (Table 3), showing the effect of these two variables on the average movement of the American crocodile; movements were more accentuated in July (low-wet season), February (brooding time), and April (hatching time). This may be reflecting a correlation between the variation of the movement patterns and seasonal periods associated with the reproductive ecology of the species. However, juveniles did not show this same movement pattern as clearly as the other groups, having their largest AMD in April (Fig 4).

These results suggest that the restricted distributions of females and wider home ranges in males, as well as the wide areas traveled by sub-adults and juveniles compared to adults and hatchlings, could be highly influenced by reproductive ecology and the precipitation cycle on Coiba [4]. Reproduction in other crocodylians (as in many other reptiles) is highly influenced by the availability of water and the precipitation cycle [7]. Precipitation on Coiba Island is strongly seasonal, having an average rainfall of 283.6 mm, ranging from 20.2 to 625.9 mm per year [53]. Most of the precipitation is limited to the so-called “wet” season between May and December. This cycle annually generates a “changing environment” that can have strong effects on movement patterns of American crocodiles in the region. These variables have not been considered or assessed in any other study of American crocodiles, so comparisons are not possible.

Several authors have reported similar results in other crocodylian species, for example in Australia [37], where males of *C*. *porosus* (salt-water crocodile) occupied larger home ranges than females in the dry season in non-tidal waterholes. Authors studying species from Africa [35] and North America [24] found that *C*. *niloticus* (Nile crocodile) and *A*. *mississippiensis* (American alligator), respectively, have large movements and home ranges associated with the wet/breeding season, highlighting the impacts of reproductive behavior and precipitation on the ways that those species utilize the surrounding physical spaces. Other authors [42,64], using telemetry methods and mark-recapture, respectively, support the contention that *C*. *porosus* males often moved considerable distances around the home range, whereas females made fewer but larger-scale movements, usually associated with visits to nest sites, consistent with our findings on *C*. *acutus*. Dissimilarities in the movement patterns and home ranges between sexes are described for *C*. *porosus* [42], possibly reflecting a consequence of the active search for females (which remain in core areas) by males during the breading season [37]. This behavior might also explain the dissimilarities we found in our study, suggesting that this might be a common behavior found for many crocodylians [65].

Using the MCP method colleagues in Florida [15] estimated an average home range of 5.6 ± 3.0 km^2^ (varying between 1.6 to 11.7 km^2^) for 10 American crocodiles (from 190 to 300 cm total length). In contrast, a pilot study by our research team [19,20] estimated average home range for 4 individuals (from 93 to 154 cm TL) on Coiba Island of 0.6 ± 1.0 km^2^, with a minimum of 0.0 km^2^ and a maximum of 2.4 km^2^. These studies did not specify what percentage (isopleth) from the MCP area these values correspond to; nevertheless, they are much larger in Florida than the areas we estimated (0.2 ± 0.2 km^2^). We cannot directly compare our results with the data obtained for *C*. *acutus* in Gatun Lake [18], as the method used to estimate home ranges was unspecified and reported was in m rather than m^2^. In Florida animals [15] AMD ranged from 0.8 to 1.4 km, somewhat greater than the AMD estimated in the current study ($\bar{x}=\;0.3\text{ km}$, ranging from 0.08 to 1.2 km). AMD values reported in the Gatun Lake study [18] are up to 0.7 km larger than in the present study, but more similar than those reported in the Florida study. The smaller area and distances traveled by American crocodiles in Coiba compared to other studies of *C*. *acutus* in mainland localities may well be the result of more limited resource availability of the insular setting.

We rejected the null hypothesis of no discernible pattern among spatial variables because we found, quantified, and statistically tested relationships among average movement distances, home ranges, age groups and sexes, influenced by seasonality and the reproductive ecology behavior of the American crocodiles in the area. We found also a clear disaggregate distribution by age groups throughout habitat types on Coiba, as has been reported by a previous study [10], where adults and hatchlings inhabited a more restricted number of habitats than juveniles and sub-adults.

Conspecific proximity (CP) analysis revealed that tagged individuals were typically at least 500 m from each other; in only 20% of these geolocations were animals 200 m or less from one another. We also found that although juveniles were never this close to adults, they were detected in the “vicinity” of sub-adults 17% of the time and of hatchlings 50% of the time. In contrast, hatchlings were around or less than 200 m from sub-adults about 4% of the time and away from adults 50% of the time. Finally, adults were up to 200 m from sub-adults around 45% of the time. These results potentially reflect a hierarchy system throughout age classes in the study area, where adults are more related in space with sub-adults, sub-adults with juveniles, and juveniles with hatchlings, respectively. The relationship between juveniles and hatchlings may be established after the parental care period, when hatchlings start to explore new areas and reduce their relative proximity to adults as they increase it with other juveniles. Predation is likely to be the most important variable that influences the relationship between juveniles and adults. However, as animals get reproductively active, more complex relationships may play a role on this hierarchy system.

Analyses of age groups [45] and size classes [59] (S1–S3 Tables; S1 Fig) represent different ways to classify individuals in order to determine the structure of a population. Both approaches have pros and cons based on the methods used to estimate them and implications of the results for efforts in management and conservation. Nevertheless, both approaches have been employed in studies monitoring American and other crocodile species [66–68]. For this reason, we think it is important to have a reference or measure of the behavior for those groups in a spatial/ecological context, which will allow researchers to visualize the movement patterns and home ranges using any of the classification methods. Finally, the current study’s results regarding home range size per age group, size class and sex should not necessarily be taken as typical for the entire species, but rather as a starting point for future research on *C*. *acutus* (and other crocodiles). Such studies will allow us to better understand how the patterns we found in Coiba may change with latitudinal in both mainland and insular localities.

Historical, current, and future extinction rates of biodiversity have been documented and modeled in many taxa with often frightening results and predictions [69,70]. These studies unanimously encourage society to reduce impacts (e.g., land cover transformation, pollution, among others) and increase ‘real’ planning (i.e., integrative and comprehensive plans) to protect and provide sustainable use of species and their habitats. However, a lack of thorough ecological knowledge for many species makes it difficult to effectively meet this challenge in many parts of the world. Furthermore, top predators face a bigger challenge due to the overlapping habitats with humans competing directly or indirectly for resources; this implies that specific spatial information is required in order to assess impacts and create inclusive management plans (top predators ecology, ethno-zoology, urban ecology, and human development plans). Our findings provide both a technical resource for developing conservation plans in Panama and a comprehensive approximation of the inter-relationship between the American crocodile and its surrounding environment, applicable across its range for baseline management and conservation planning. They can serve as a valuable starting point relative to understanding crocodile spatial ecology in other areas (e.g., mainland populations).

## Supporting Information

S1 Fig Home range and utilization distribution. Utilization distribution per size classes using Kernel Density Estimation (KDE).
(TIF)

S1 Table Trajectories, movement distances, and speed of the American crocodile by size class. Number of path trajectories (N), time average between locations (TAG), average movement distance (AMD), and average movement speed (AMS) followed by American crocodiles in Coiba Island per size class.
(DOCX)

S2 Table Conspecific proximity among American crocodiles by size class. Number of observations, percent of geolocations closer than 200 m, and average conspecific proximity (ACP) within a time overlapping of 6 h estimated for 24 American crocodiles followed in Coiba Island from 2010 to 2013 divided by size class.
(DOCX)

S3 Table Home range and utilization distribution of the American crocodiles by size class. Home range and utilization distribution of the American crocodiles on Coiba Island estimated via Minimum Convex Polygon (MCP), Kernel Density Estimation (KDE), and Local Convex Hull—adaptive (aLoCoH). These values were estimated based on the average by individual + SD, reporting the maximum and minimum values obtained. Data for all individuals and averages divided by size classes are reported. Analyses were made including all data on all individuals and using isopleths at 50% and 95%.
(DOCX)
