# Supplementary figures and images for: Spatial Ecology of the American Crocodile in a Tropical Pacific Island in Central America
Source: PLoS One. 2016 Jun 9;11(6):e0157152. doi: 10.1371/journal.pone.0157152 (PMC4900666; doi:10.1371/journal.pone.0157152)

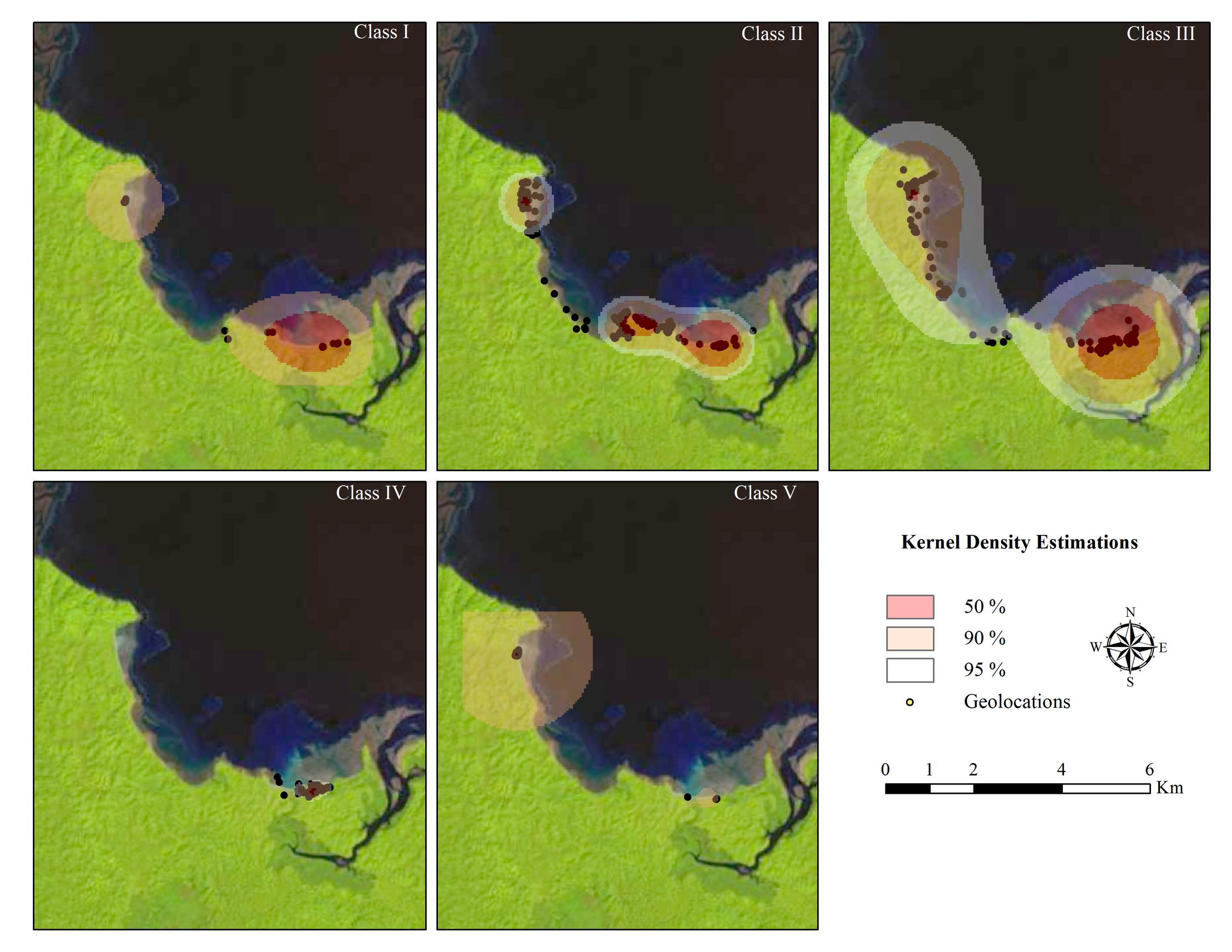

Supplement: S1 Fig — Utilization distribution per size classes using Kernel Density Estimation (KDE). (TIF) [file pone.0157152.s001.tif]
