# Supplementary material for: Spatial Ecology of the American Crocodile in a Tropical Pacific Island in Central America
Source: PLoS One. 2016 Jun 9;11(6):e0157152. doi: 10.1371/journal.pone.0157152 (PMC4900666; doi:10.1371/journal.pone.0157152)
Supplement: S1 Table — Number of path trajectories (N), time average between locations (TAG), average movement distance (AMD), and average movement speed (AMS) followed by American crocodiles in Coiba Island per size class. (DOCX) [file pone.0157152.s002.docx]

S1 Table.

| **Groups** | **# of individuals** | **N** | **TAG (h) max-min** | **AMD (m) max-min** | **SP (km/h) max-min** |
| --- | --- | --- | --- | --- | --- |
| Class V | 1 | 13 | 141 (3-1319) | 907 (8-5607) | 0.02 (0.0-0.24) |
| Class IV | 4 | 60 | 293 (4-1653) | 201 (2-1055) | 0.01 (0.0-0.12) |
| Class III | 7 | 206 | 203 (2-3833) | 323 (3-4337) | 0.01 (0.0-0.34) |
| Class II | 12 | 191 | 180 (1-2455) | 276 (2-5807) | 0.01 (0.0-0.26) |
