# Supplementary material for: Spatial Ecology of the American Crocodile in a Tropical Pacific Island in Central America
Source: PLoS One. 2016 Jun 9;11(6):e0157152. doi: 10.1371/journal.pone.0157152 (PMC4900666; doi:10.1371/journal.pone.0157152)
Supplement: S2 Table — Number of observations, percent of geolocations closer than 200 m, and average conspecific proximity (ACP) within a time overlapping of 6 h estimated for 24 American crocodiles followed in Coiba Island from 2010 to 2013 divided by size class. (DOCX) [file pone.0157152.s003.docx]

S2 Table.

|  | **N** | **Up to 200 m (%)** | **ACP (m)** |
| --- | --- | --- | --- |
| Class I to II | 11 | 9 | 2,616.26 ± 2,178.78 |
| Class I to III | 17 | 6 | 1,756.72 ± 1,868.94 |
| Class I to IV | 4 | 100 | 63.28 ± 58.34 |
| Class I to V | - | - | - |
| Class II to V | 29 | 21 | 1,639.70 ± 1630.33 |
| Class II to IV | 59 | 57 | 673.49 ± 1,309.55 |
| Class II to III | 245 | 11 | 1,665.01 ± 1,810.01 |
| Class III to IV | 88 | 49 | 876.51 ± 1,571.75 |
| Class III to V | 25 | 0 | 2,562.83 ± 1,767.77 |
| Class IV to V | 2 | 0 | 5,619.73 |
