# Supplementary material for: Spatial Ecology of the American Crocodile in a Tropical Pacific Island in Central America
Source: PLoS One. 2016 Jun 9;11(6):e0157152. doi: 10.1371/journal.pone.0157152 (PMC4900666; doi:10.1371/journal.pone.0157152)
Supplement: S3 Table — Home range and utilization distribution of the American crocodiles on Coiba Island estimated via Minimum Convex Polygon (MCP), Kernel Density Estimation (KDE), and Local Convex Hull—adaptive (aLoCoH). These values were estimated based on the average by individual + SD, reporting the maximum and minimum values obtained. Data for all individuals and averages divided by size classes are reported. Analyses were made including all data on all individuals and using isopleths at 50% and 95%. (DOCX) [file pone.0157152.s004.docx]

S3 Table.

|  | **50% (km2)** | | | **95% (km2)** | | |
| --- | --- | --- | --- | --- | --- | --- |
|  | **MCP** | **KDE** | **aLoCoH** | **MCP** | **KDE** | **aLoCoH** |
| V | 0.00 | 0.00 | - | 1.34 | 7.24 | - |
| IV | 0.01 | 0.01 | 0.01 | 0.12 | 0.29 | 0.09 |
| III | 8.42 | 2.54 | 0.27 | 10.31 | 23.98 | 2.35 |
| II | 2.15 | 1.46 | 0.33 | 8.87 | 5.97 | 1.19 |
| I | 0.44 | 1.98 | 0.17 | 4.34 | 8.09 | 0.31 |
